# 

**Published:** 1897-05

**Authors:** 


### CONTENTS.

COMMUNICATIONS.

What Can We Do to Increase the Attendance at Our Dental Societies? 209
Education of the People about the Teeth ........ 217

ELECTRICITY IN DENTISTRY.

Electricity in Dentistry ........ 220
Electrolysis in Dentistry ........ 221
Cataphoresis and Anaphoresis ........ 224

SELECTIONS.

Exostosis ........ 226
Removal of Deposits Upon Teeth ........ 233
Oral Surgery—Theory and Results ........ 243
Cocaine—Its Use and Abuse ........ 245
Formalin ........ 249
A Bright Lad ........ 251
Care of the Teeth ........ 252
Michigan Dental Association Annual Meeting ........ 253

DENTISTS IN THE ARMY.

Employment of Dentists in the Army ........ 253

EDITORIAL.

Biographical ........ 256
Illinois State Dental Society ........ 257
Dental Societies—Directory ........ 257
Death of Dr. Frank Abbott and Dr. J. C. Story ........ 258
Catching's Compendium of Practical Dentistry ........ 258
Programme of the Dental and Oral Section of the American Medical Association ........ 259

QUESTIONS.

Questions Submitted for Discussion by Local Societies—Formulated by the Committee Appointed by the American Dental Association 260

